# Genetic characterization of a rare case of pheochromocytoma in a pulmonary transplant patient

**DOI:** 10.3389/fendo.2024.1481906

**Published:** 2025-02-06

**Authors:** Stéfanie Parisien-La Salle, Florence Perreault, Gilles Corbeil, Julie Morisset, Charles Poirier, Catherine Beauregard, Agnès Räkel, Marjorie Labrecque, Martine Tétreault, Christian Cohade, Pasquale Ferraro, Isabelle Bourdeau

**Affiliations:** ^1^ Division of Endocrinology, Department of Medicine, Centre hospitalier de l’Université de Montréal Research Center (CRCHUM), Montreal, QC, Canada; ^2^ Division of Respirology, Department of Medicine, Centre hospitalier de l’Université de Montréal Research Center (CRCHUM), Montreal, QC, Canada; ^3^ Neuroscience Division, Centre de Recherche du Centre Hospitalier de l’Université de Montréal (CRCHUM), Montreal, QC, Canada; ^4^ Department of Neurosciences, University of Montreal, Montreal, QC, Canada; ^5^ Division of Nuclear Medicine, Department of Radiology, Centre hospitalier de l’Université de Montréal (CHUM), Montreal, QC, Canada; ^6^ Division of Thoracic Surgery, Department of Surgery, Centre hospitalier de l’Université de Montréal Research Center (CRCHUM), Montreal, QC, Canada

**Keywords:** pheochromocytoma, hypoxia, genetics, RNA-sequencing, transplant

## Abstract

**Background:**

Pheochromocytomas (PCCs) and paragangliomas (PGLs) (PPGLs) are rare tumours arising from the chromaffin cells. There is evidence suggesting a link between hypoxemia and PPGLs. Chronic hypoxia can lead to gain of function somatic variants in the *EPAS1* gene that encodes for hypoxia-inducible factor 2-alpha (HIF-2α), involved in PPGL tumorigenesis.

**Objective:**

To describe a rare case of PCC in a pulmonary transplant patient and characterize the tumour’s genetic background.

**Clinical Case:**

A 47 year-old man underwent a lung transplant for chronic obstructive pulmonary disease associated with alpha-1 antitrypsin deficiency. He required home oxygen therapy for 3 years prior to transplant. Nineteen years after transplant, a CT-scan revealed a 5.8 cm x 3.9 cm heterogeneous right adrenal mass (HU of 7). Initial assessments indicated elevated 24-hour urinary catecholamines. Consequently, the patient underwent laparoscopic right adrenalectomy, confirming the PCC diagnosis.

**Genetic studies:**

1) Germline PPGL multigene panel: After consent, the patient underwent a panel of 14 susceptibility genes for PPGLs that revealed no pathogenic variants. 2) Somatic genetic analysis for *EPAS1* gene found no variants. However, tumoral RNA sequencing unveiled activation of the HIF pathway.

**Conclusion:**

We describe a rare case of PCC in a pulmonary transplant recipient, with genetic analyses showing no germline pathogenic variants and no somatic variants in the *EPAS1* gene. RNA sequencing highlighted HIF pathway activation and angiogenic implications. Further research is necessary to elucidate the genetic and molecular mechanisms underlying PCCs in this specific case and determine its link with hypoxemia in the context of pulmonary disease.

## Introduction

Pheochromocytomas (PCCs) and paragangliomas (PGLs) (PPGLs) are rare neuroendocrine tumours arising from the adrenal medulla or along the sympathetic/parasympathetic ganglia chains (1). PPGLs are the most heritable tumours, with 30–35% of patients of European descent having a germline variant in a known susceptibility gene (2). More than 20 susceptibility genes for PPGLs have been described (germline and somatic) and are divided into 3 clusters: pseudohypoxia cluster 1 (1A and 1B), kinase-signalling cluster 2, and Wnt signalling cluster 3 (2, 3).

Several studies have shown that different hypoxemic states can be linked to the pathogenesis of PPGLs including high altitude, congenital heart anomalies or a pseudohypoxic state due to variants in cluster 1 genes (2–6). Indeed, tumours with pathogenic variants in cluster 1 genes display a stabilization and accumulation of hypoxia induced factor (HIF), leading to angiogenesis and tumour invasion (2, 6). Chronic hypoxia due to high altitude has been shown to be associated with a higher incidence of head and neck paragangliomas (HNPGLs) (4). Genetic analysis of a small group of high altitude HNPGLs yielded negative results for pathogenic variants in *SDHB* and *SDHD* genes (7). Recently, a meta-regression analysis was performed and showed a higher incidence of PPGLs in areas of higher altitude (8). However, this study was criticized due to the lack of inclusion of studies and heterogeneity within the studies (9). Another hypoxia inducing pathology with an association to PPGLs is cyanotic congenital heart disease (CCHD) (5). In a study by Opotowsky et al., CCHD hospitalised patients had an odds ratio of 6 for developing PPGLs compared to patients without CCHD (5). Moreover, gain of function somatic variants in the *EPAS1* gene that encodes for hypoxia-inducible factor 2-alpha (HIF2α) were identified in CCHD associated PPGLs (10). In a study by Vaidya et al., 4/5 (80%) CCHD related PPGLs harboured a somatic variant in codon 530 or 531 in exon 12 of *EPAS1*. These residues code for the “oxygen degradation domain of HIF2α” (10). Out of the 5 patients for whom germline DNA was available (4/5), no pathogenic variants were identified in *EPAS1* or the 10 PPGL susceptibility genes that were tested. This being said, it remains uncertain whether a hypoxemic state in other conditions, such as pulmonary diseases, could also contribute to the development of PPGLs. Here, we report a rare occurrence of PCC in a pulmonary transplant recipient, providing genetic characterization of the tumour.

## Case description

We report the case of a 66 year-old man who underwent a unilateral lung transplant at the age of 47 for chronic obstructive pulmonary disease associated with alpha-1 antitrypsin deficiency. Prior to transplant, his forced expiratory volume in 1 second (FEV1) was 1.49 L (11% of his predicted value) and his forced vital capacity (FVC) was 1.45 L (25% of his predicted value). His exercise test showed a VO2 max of 0.7 L/min (21% of predicted value). At room air, his 6 min walk test results showed a total distance 150.4 m with a saturation of 89% at 73% of max heart rate. He required home oxygen therapy for 3 years prior to transplant. Following transplant, the patient was diagnosed with stage 4 chronic kidney disease, hypertension under dual therapy and paroxysmal atrial tachycardia and post-transplantation diabetes mellitus (PTDM). Patient’s family history was non-contributing. Hypoxia resolved after pulmonary transplant, with the patient achieving normal ambient air saturation and no longer requiring supplemental oxygen.

Eleven years after transplant, a follow-up chest CT scan described a right adrenal nodule of 1.9 x 0.8 cm. Two years later, on a chest CT scan, the nodule grew and was measured at 2.4 x 1.1 cm. No density measurements were described. Urinary catecholamines were ordered and urinary metanephrines were mildly elevated (norepinephrine 211 nmol/d (N <650), epinephrine <10 nmol/d (N <145), dopamine 867 nmol/d (N <4520), normetanephrines 507 nmol/d (N <600) and metanephrines 613 nmol/d (N <370)). However, the test result was lost to follow-up.

**Figure 1:**
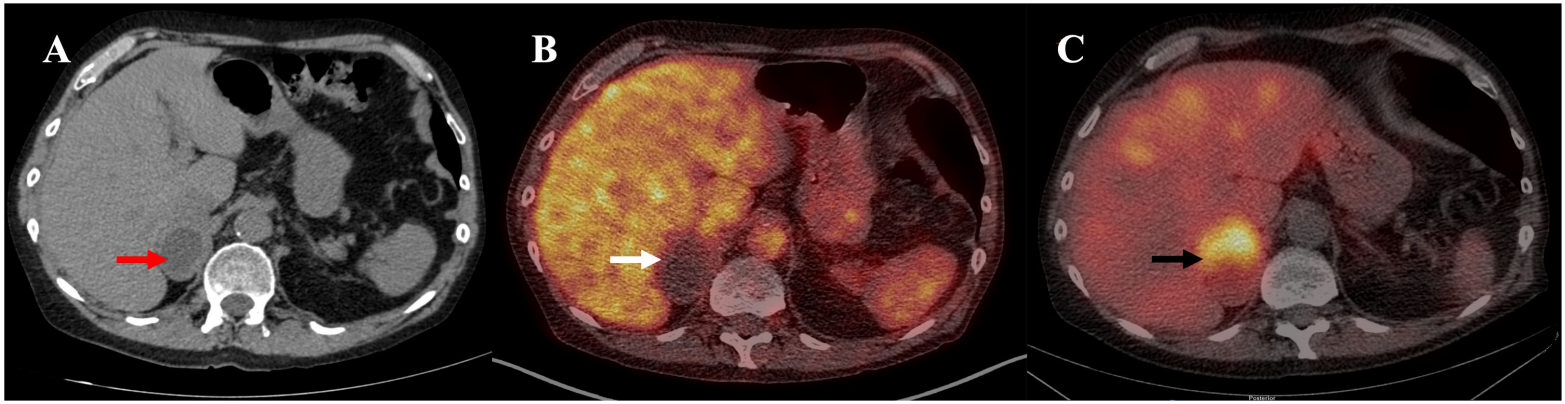
**(A)** Unenhanced CT: heterogenous 5.8 cm x 3.9 cm right adrenal mass with a density of 7 Hounsfield Units (HU) (red arrow). **(B)** ^18^F-FDG PET/CT, fused image: inactive 5.8 x 4.0 x 4.1cm right adrenal mass (white arrow). **(C)** ^123^I-MIBG SPECT/CT, fused image: right adrenal mass with intense MIBG uptake (black arrow).

Nineteen years after transplant, a follow-up thoracic CT scan revealed the growth of the right adrenal nodule and described it as a heterogenous 5.8 cm x 3.9 cm mass with a density of 7 Hounsfield Units (HU) (**Figure 1**). An adrenal panel was ordered, and the diagnosis of PCC was confirmed by the 24 h urinary catecholamines (norepinephrine 713 nmol/d (N <650), epinephrine 588 nmol/d (N <145), dopamine 205 nmol/d (N <4520), normetanephrines 900 nmol/d (N <600) and metanephrines 1191 nmol/d (N <370)). Chromogranin A was also elevated (3297 ng/mL (N <104)), as were plasmatic metanephrines (6.99 nmol/L (n <0.48)) and normetanephrines (5.79 nmol/L (N <1.20)). The rest of the hormonal adrenal panel was normal (**Table 1**). The adrenal mass showed no uptake at ^18^F-FDG PET/CT imaging but fixation on MIBG scintigraphy (**Figure 1**). The patient reported rare episodes of diaphoresis, but no palpitations or headaches. He underwent a laparoscopic right adrenalectomy. The pathology report confirmed a 6.2 cm PCC with capsular invasion, micro foci of invasion of the periadrenal fatty tissue, pleomorphic cells, cellular monotony and large nests (PASS score 8-10). Staining for SDHB protein was intact and ki-67 proliferation index was <1%. Two years following PCC resection, patient’s diabetes and hypertension remained unchanged. Imaging and biochemical testing showed no signs of PCC recurrence.

**Table 1:** Hormonal Adrenal panel results.

	Results	Normal range
**Renin** (ng/L)	42	–
**Aldosterone** (pmol/L)	813	–
**Aldosterone to renin ratio**	19.4	N <85
**1 mg dexamethasone test**	Patient chronically on prednisone	
**24h Urinary Catecholamines**		
Norepinephrine (nmol/d)	**713**	N <650
Epinephrine (nmol/d)	**588**	N <145
Dopamine (nmol/d)	205	N < 4520
Normetanephrines (nmol/d)	**900**	N < 600
Metanephrines (nmol/d)	**1191**	N < 370
**Plasmatic normetanephrines** (nmol/L)	**5.79**	N < 1.20
**Plasmatic metanephrines** (nmol/L)	**6.99**	N <0.48
**Plasmatic 3-methoxytyramine** (nmol/L)	0.08	N <0.16
**Chromogranin A** (ng/mL)	**> 800**	N <82

*Bold indicates abnormal values.

## Methods

### Biochemistry

Measurement of free urinary catecholamines and metanephrines:

Samples from 24-hour urine collections, with the addition of 3,4-dihydroxybenzylamine as an internal standard, were adjusted to pH 6.5 prior to off-line solid phase extraction on ion-exchange mini-columns (Bio-Rad). Catecholamines and free metanephrines were separated on a Kinetex LC C18 100A column (150 x 4.6 mm, 5 µm; Phenomenex) using isocratic elution on an Ultimate 3000 HPLC system (Thermo Scientific) coupled to a Coulochem III electrochemical detector (ESA).

### Measurement of free plasma metanephrines

Plasma samples, with the addition of deuterated internal standards, were subjected to on-line SPE extraction (CHRO SPE Polymer WCX cartridges), followed by HPLC separation of free metanephrines on an Atlantis HILIC silica column (100 A, 2.1 X 50 mm, 3 µm; Waters) and detection with tandem mass spectrometry on a Chronect Symbiosis-TSQ Quantis LC-MSMS system (Spark Holland/Thermo).

### Germline PPGL multigene panel

The patient underwent genetic counselling, and a multigene panel was ordered that included 14 susceptibility genes: *SDHA, SDHAF2, SDHB, SDHC, SDHD, RET, VHL, FH, NF1, MAX, TMEM127, EGLN1, KIF1B, MEN1* genes (Invitae, San Francisco, CA). Gene sequencing and deletion/duplication analysis were performed using NGS technology for all genes except for *SDHA*, which was not evaluated for deletion/duplication. Written consent form was obtained from the patient before genetic analysis. Leucocyte DNA was extracted from whole blood cells.

### Somatic genetic analysis for *EPAS1* gene

Tumoral DNA was extracted from FFPE tissues as described previously (11). Exons 9, 12 and 16 of the *EPAS1* gene were studied by Sanger Sequencing (**Supplementary Table S1**). These 3 exons were chosen as they are sites for the three regulatory hydroxylation domains (10). The amplicons were directly sequenced using the Applied Biosystems 3730xl DNA Analyzer (The Centre d’expertise et de services Génome Québec CHU-Sainte-Justine).

### RNA sequencing

RNA sequencing of patient leukocyte and tumoral RNA was performed (Research Center CHU Québec) and a commercially available pool of human adrenal total RNA (Clontech, Mountain View, CA) was used as a reference sample for tumoral RNA. Five additional patients with PPGLs, who were not exposed to chronic hypoxia and tested negative on the multigene panel for PPGLs, were also included as a combined control group (RNA and tumoral RNA).

## Results

### Germline PPGL multigene panel

No pathogenic variants or variants of unknown significance were detected in the multigene panel, which comprised 14 susceptibility genes for PPGLs.

### Somatic genetic analysis for *EPAS1* gene

No pathogenic variants were identified in all three tested exons of the *EPAS1* gene.

### Leukocyte RNA sequencing

Compared to leucocyte RNA of a pool of five patients with PPGL who did not undergo chronic hypoxia and had a negative multigene panel, our patient’s leucocyte RNA showed an overexpression of *EPAS1 (*or *HIF2α)* (log2 fold change: 5.69 *p=1.26E-08*). *EPAS1* is implicated in multiple pathways, including ‘Pathways in cancer’ (hsa05200). We conducted KEGG pathway enrichment analysis using DAVID software (12). Notably, our analysis revealed that our top upregulated genes (452 genes with p < 0.05) are significantly associated with this pathway, showing a p-value of *0.0017* and a fold enrichment of 1.9. Other upregulated genes in this pathway included *HIF1α* (log2 fold change: 3.80 *p=0.000145*) and *VEGF-C* (log2 fold change: 2.46 *p=0.0140*). Interestingly, *EGLN2*, a *HIF1α* inhibitor, was significantly downregulated (log2 fold change: -2.65 *p=0.00813)* (**Table 2A**).

**Table 2A:** Leucocyte RNA sequencing.

Genes	Leucocyte RNA sequencing: Patient leucocyte RNA VS pooled leucocyte RNA of 5 patients with PPGLs without chronic hypoxia	
	Log2 fold change	p-value
*EPAS1*	5.69	1.26E-08
*HIF1α*	3.80	0.000145
*VEGF-C*	2.46	0.0140

**Table 2B:** Tumoral RNA sequencing.

Genes	Tumoral RNA sequencing: patient tumoral RNA VS commercially available pool of human adrenal total RNA (Clontech, Mountain View, CA)		Tumoral RNA sequencing: patient tumoral RNA VS pooled tumoral RNA of 5 patients with PPGLs without chronic hypoxia	
	Log2 fold change	p-value	Log2 fold change	p-value
*EPAS1*	-0.85	0.396	-0.22	0.828
*VEGF-A*	2.08	0.0371	2.90	0.00371
*FLT1*	5.83	5.39E^-9^	2.33	0.02
*SERPRINE1*	5.14	2.7E^-7^	4.65	3.25E^-6^

### Tumoral RNA sequencing

*EPAS1* was not found to be significantly modified in tumour tissue comparisons in either human adrenal total RNA (log2 fold change: -0.85 *p=0.396*) or tumoral RNA of other PPGLs (log2 fold change: -0.22 *p=0.828*).

However, in the *HIF-1* KEGG signalling pathway (hsa04066), three genes implicated in angiogenesis were highly upregulated when compared to human adrenal total RNA and other PPGL tumoral RNA: *VEGF-A* (human adrenal total RNA: log2 fold change: 2.08 *p=0.0371;* other *PPGL* tumoral RNA: log2 fold change: 2.90 *p=0.00371), FLT1 (or VEGFR1)* (human adrenal total RNA: log2 fold change: 5.83 *p=5.39E^-9^; other PPGL* tumoral RNA: log2 fold change: 2.33 *p=0.02), SERPRINE1* (human adrenal total RNA: log2 fold change: 5.14 *p=2.7E^-7^; other PPGL* tumoral RNA: log2 fold change: 4.65 *p=3.25E^-6^)* (**Table 2B**).

## Discussion

This case illustrates the rare association between a lung transplant patient and PCC. As described above, hypoxia plays a significant role in the pathogenesis of PPGLs. Our case is of particular interest because it describes a patient with chronic hypoxia not induced by CCHD or altitude who developed a PCC.

The timing and degree of hypoxia associated with PPGLs remains unknown. In a study by Opotowsky et al., the mean duration of hypoxia for PPGLs associated with CCHD was 20 years (range 1–57 y) (5). In a review of patients with CCHD who underwent a Fontan procedure and were diagnosed with PPGLs, cyanosis duration ranged from 15 months to 13 years (13). It remains unclear whether the timing or degree of hypoxia plays a part in the pathogenesis. Interestingly, our patient did not present a known pathogenic variant in the germline PPGL multigene panel. This is often the case in patients with CCHD related PPGLs (5, 14). One patient with CCHD and a multifocal recurrent PGL harboured an *SDHB* pathogenic missense variant (11). Also, our patient did not present with an *EPAS1* somatic variant, that is often found in CCHD associated PPGLs (10).

To explore if the patient’s chronic hypoxia played a role in the pathogenesis of his PCC, we performed RNA sequencing. We found activation of HIF pathway at various levels. Leukocyte RNA showed overexpression of *EPAS1*, *HIF1α* and *VEGF-C*, while tumoral RNA sequencing revealed overexpression of genes in the HIF pathway implicated in angiogenesis (*VEGF-A*, *FLT1*, *SERPINE1*). Interestingly, an *in vitro* study showed that *VHL* knockdown PC12 cells also overexpress *SERPINE1* (15). In a recent study, PC12 cells (Rat pheochromocytoma cells) were exposed to 20 recurrent hypoxia cycles (PC12 Z20) (16). RNA sequencing demonstrated an upregulation of HIF2α (Hypoxia-Inducible Factor 2 Alpha) in these cells. The authors observe that: “PC12 Z20 cells showed a higher growth rate, and the migration and adhesion capacity were significantly increased compared with control cells.” Although these findings are interesting, they do not clearly prove that hypoxia was the driving factor for the development of the PCC.

Another driving factor for the patient’s PCC could have been his transplant. Transplant patients have higher malignancy rates than the general population (17). Chronic immunosuppressive therapy following a transplant can play a role in the development of malignancies (17). Lung transplant patients receive more immunosuppression than other soft organ transplants (17). The most frequent malignancies following lung transplant are skin cancer, lung cancer and post-transplant lymphoproliferative disease (17). In the literature, we identified two cases of PCCs in lung transplant patients (18, 19). The first case is a 44-year-old patient with heart, lung and renal transplantation secondary to cystic fibrosis and diabetes. She was diagnosed with a PCC following multiple episodes of hypertension and tachycardia. No genetic analysis was done on the tumor or germline DNA and no information was given on duration of hypoxia before diagnosis of PCC or the timing of the diagnosis after the transplant (18). The second case is a 25-year-old man with double lung transplant for cystic fibrosis who was diagnosed with a PCC after investigations for resistant hypertension. Again, no genetic analysis was reported on the tumor and germline DNA results were pending and no information was given on duration of hypoxia or timing of transplant in regards to the diagnosis of the PCC (19).

Another interesting point of this case is the radiological description of the adrenal lesion. Indeed, the patient presented with a heterogeneous PCC that had an overall density of 7 HU on a CT-scan without contrast. This is a very rare finding. So rare, that some guidelines do not recommend to screen biochemically adrenal incidentalomas with ≤ 10 HU for PCCs (20, 21). However, an important nuance is that these guidelines also recommend that a heterogeneous mass should be considered for biochemical testing (20, 21). In a large retrospective multicentre study, the sensitivity of the 10 HU cut-off for identifying a PCC was 99.6% (22). The only patient who presented with a PCC of -4 HU also had an ACTH ectopic co-secretion by the PCC, which could lead to a false negative due to higher cholesterol content within the tumor (22).

Finally, from a clinical standpoint, the patient’s diabetes and hypertension showed no improvement following PCC resection. This persistence is likely due to several contributing factors: the extended duration between the initial diagnosis and the surgery, which allowed for the establishment of irreversible metabolic and vascular changes (23); the ongoing use of diabetogenic immunosuppressive therapy required for post-transplant management, perpetuating hyperglycemia (24); and the progression of advanced chronic kidney disease, which exacerbates and sustains hypertension through mechanisms such as fluid retention, altered renin-angiotensin-aldosterone system activity, and sympathetic overactivity (25).

In sum, we report a rare case of PCC in a pulmonary transplant patient. Our genetic analyses demonstrated the absence of a pathogenic germline variant in a multigene panel of 14 genes related to PPGL and the absence of a somatic variant in the hypoxemia related gene *EPAS1*. RNA sequencing did demonstrate activation of the HIF pathway and implication of angiogenesis; however, further work is needed to better understand the genetic and molecular events leading to PCC in this specific case and determine its relation with hypoxemia in the context of pulmonary disease.

## Data Availability

The raw data supporting the conclusions of this article will be made available by the authors, without undue reservation.
