# Supplementary material for: Genetic characterization of a rare case of pheochromocytoma in a pulmonary transplant patient
Source: Front Endocrinol (Lausanne). 2025 Feb 6;15:1481906. doi: 10.3389/fendo.2024.1481906 (PMC11839448; doi:10.3389/fendo.2024.1481906)
Supplement: Supplementary file 1 [file Table1.docx]

**Supplemental Table 1: Primers for *EPAS1* amplification**

|  | **Forward Primer** | **Reverse Primer** |
| --- | --- | --- |
| **Exon 9** | ATGCCTGGAGTCCTACCCAT | AAGAAAAAGCTGCAGCCAGG |
| **Exon 12** | CTGCAGGAGCTGAGTTGGAA | TACTAGTGGGTGCCTCTCGG |
| **Exon 16** | GGGACAGACACCACTGAAGG | CTTGGTGACCTGGGCAAGTC |
